# Bis(2,2′-bipyridine)bis­{μ_3_-*cis*-*N*-(2-carboxyl­atophen­yl)-*N*′-[3-(dimethyl­amino)prop­yl]oxamidato(3−)}­bis(per­chlorato)­tetra­nickel(II) methanol disolvate

**DOI:** 10.1107/S160053680905017X

**Published:** 2009-11-28

**Authors:** Chunliang Tian, Zhongjun Gao

**Affiliations:** a Deapartment of Chemistry, Jining University, Shandong 273155, People’s Republic of China

## Abstract

In the title methanol disolvate complex, [Ni_4_(C_14_H_16_N_3_O_4_)_2_(ClO_4_)_2_(C_10_H_8_N_2_)_2_]·2CH_3_OH, the neutral tetra­nickel(II) system lies on a centre of inversion. The polyhedron around each Ni(II) atom is a square pyramid. The separations of the Ni atoms bridged by the oxamide and carboxyl groups are 5.227 (9) and 5.268 (6) Å, respectively. In the crystal structure, a two-dimensional supramolecular network structure involving O—H⋯O and C—H⋯O hydrogen bonding is observed.

## Related literature

For a related structure, see: Tao *et al.* (2003 ▶).
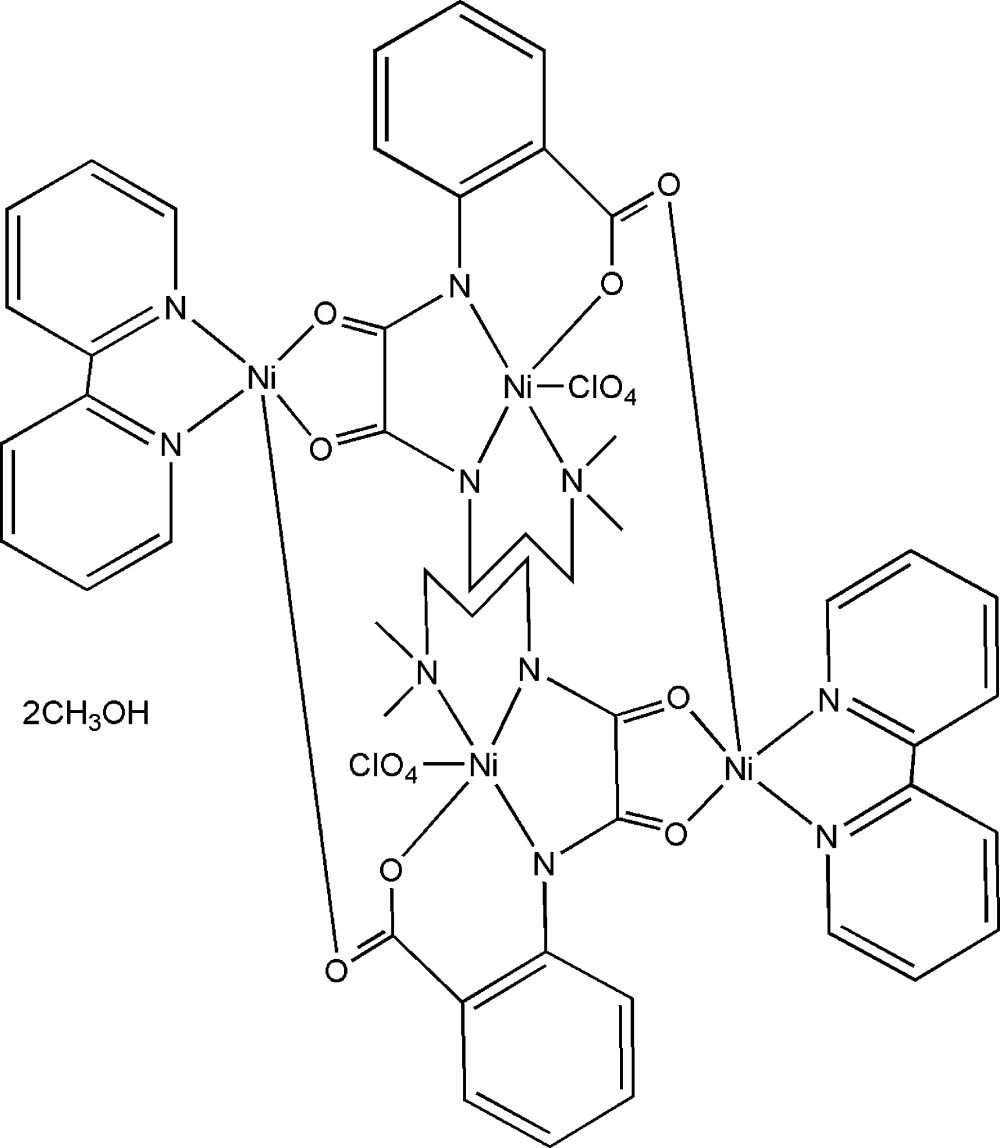


## Experimental

### 

#### Crystal data

[Ni_4_(C_14_H_16_N_3_O_4_)_2_(ClO_4_)_2_(C_10_H_8_N_2_)_2_]
*M* *_r_* = 1390.79
Triclinic, 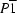

*a* = 10.854 (4) Å
*b* = 11.309 (4) Å
*c* = 12.728 (5) Å
α = 67.724 (4)°
β = 73.357 (4)°
γ = 75.411 (4)°
*V* = 1367.0 (9) Å^3^
*Z* = 1
Mo *K*α radiation
μ = 1.54 mm^−1^
*T* = 298 K
0.21 × 0.16 × 0.14 mm

#### Data collection

Bruker SMART CCD diffractometer
Absorption correction: multi-scan (*SADABS*; Sheldrick, 1996 ▶) *T*_min_ = 0.738, *T*_max_ = 0.814
7296 measured reflections
4850 independent reflections
3963 reflections with *I* > 2σ(*I*)
*R*_int_ = 0.015

#### Refinement

*R*[*F* ^2^ > 2σ(*F* ^2^)] = 0.032
*wR*(*F* ^2^) = 0.087
*S* = 1.04
4850 reflections
383 parameters
H-atom parameters constrained
Δρ_max_ = 0.33 e Å^−3^
Δρ_min_ = −0.56 e Å^−3^

### 

Data collection: *SMART* (Bruker, 1998 ▶); cell refinement: *SAINT* (Bruker, 1998 ▶); data reduction: *SAINT*; program(s) used to solve structure: *SHELXS97* (Sheldrick, 2008 ▶); program(s) used to refine structure: *SHELXL97* (Sheldrick, 2008 ▶); molecular graphics: *SHELXTL* (Sheldrick, 2008 ▶); software used to prepare material for publication: *SHELXL97* and *PLATON* (Spek, 2009 ▶).

## Supplementary Material

Crystal structure: contains datablocks I, global. DOI: 10.1107/S160053680905017X/pv2236sup1.cif

Structure factors: contains datablocks I. DOI: 10.1107/S160053680905017X/pv2236Isup2.hkl

Additional supplementary materials:  crystallographic information; 3D view; checkCIF report

## Figures and Tables

**Table 1:** Hydrogen-bond geometry (Å, °)

*D*—H⋯*A*	*D*—H	H⋯*A*	*D*⋯*A*	*D*—H⋯*A*
O9—H9⋯O1^i^	0.82	2.73	3.190 (4)	118
O9—H9⋯O2^i^	0.82	2.06	2.870 (4)	170
C4—H4⋯O9^ii^	0.93	2.52	3.387 (5)	155
C13—H13*C*⋯O9^iii^	0.96	2.58	3.451 (5)	151
C3—H3⋯O2	0.93	2.41	2.744 (4)	101
C6—H6⋯O3	0.93	2.22	2.812 (3)	121
C10—H10*B*⋯O4	0.97	2.43	2.775 (4)	100
C13—H13*A*⋯O1	0.96	2.39	2.899 (4)	113
C13—H13*B*⋯O5	0.96	2.47	3.186 (4)	131
C14—H14*C*⋯O1	0.96	2.58	3.074 (5)	112
C24—H24⋯O4	0.93	2.59	3.065 (4)	112

Symmetry codes: (i) 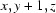; (ii) 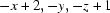; (iii) 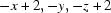.

**Fig. 1.**
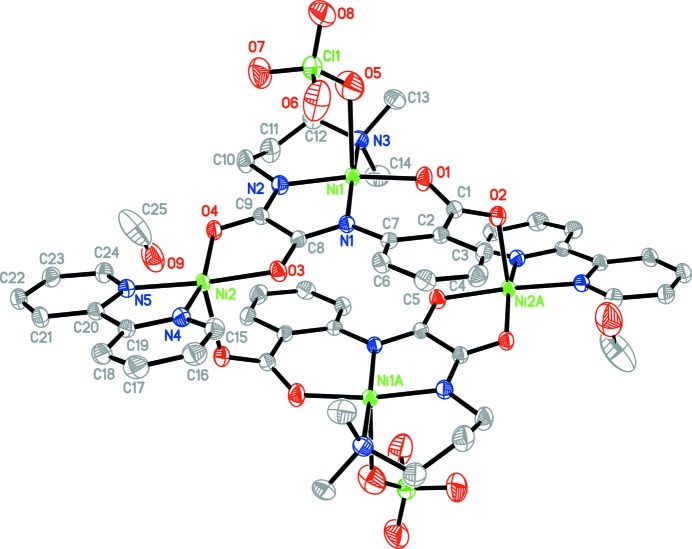
The molecular structure of (I) with 30% displacement ellipsoids (H atoms omitted for clarity).

## supplementary crystallographic information

### Comment

The title compound (Fig. 1), is a tetranuclear nickel(II) complex. Its asymmetric unit is composed of a cis-oxamido bridged dinuclear nickel complex and a molecule of methanol solvate. Through carboxyl bridges, two dinuclear units are assembled to form a circular tetranuclear system lying about an inversion center. The cis-oxamido group coordinates to Ni1 and Ni2 in a usual mode with the bite angles of 83.53 (6) and 85.14 (11) °, respectively. Both Ni1 and Ni2 atoms are in square-pyramidal coordination geometries. The maximum displacement from the least-square plane defined by N1, N2, N3 and O1, is 0.0400 (11) Å for N1 and the Ni1 atom lies 0.1168 (12) Å out of this plane. The apical position of Ni1 is occupied by O5 with the Ni1—O5 bond length of 2.636 (8) Å. Ni2 atom coordinates to the exo-cis oxygen atoms of oxamido ligand (O3 and O4). The two oxygen atoms and the nitrogen atoms (N4 and N5) of bipyridine ligand complete the basal plane, from which the maximum deviations is 0.1311 (6) Å. The apical site is occupied by a carboxyl oxygen atom (O2^i^) with Ni2—O2^i^ length of 2.276 (2) Å. The Ni—N bond lenghts in (I) (Table 1), lie in the range 1.945 (2)-2.067 (2)Å and are close to the corresponding bond lenghts reported in a nickel complex (Tao *et al.*, 2003).

In the crystal, neutral tetranuclear complexes and methanol molecules are connected by classcial O—H···O and non-classical C—H···O hydrogen bonds into a two-dimensional network (Table 1).

### Experimental

A methanol solution (5 ml) of Ni(ClO_4_)_2_^.^6H_2_O (0.732 g, 2 mmol) was added slowly into a methanol solution (5 ml) containing N-benzyl-N'-(3-amino-3-dimethylpropyl)oxamide (1 mmol, 0.293 g) and sodium ethoxide (0.204 g, 3 mmol). The mixture was stirred quickly for 1 h, then an aqueous solution (5 ml) of 2,2'-bipyridine (0.156 g, 1 mmol) was added dropwise into the mixture. The reaction solution was heated at 343 K with stirring for 8h. The resulting solution was filtered and the filtrate was kept at room temperature. Green crystals suitable for X-ray analysis were obtained from the filtrate by slow evaporation for about one week.

### Refinement

H atoms were positioned geometrically [0.93 (CH), 0.97 (CH_2_), 0.96 (CH_3_) and 0.82 (OH)Å] and constrained to ride on their parent atoms with *U*_iso_(H) =1.2(1.5 for methyl)*U*_eq_(C/N).

### Crystal data

**Table:**

[Ni_4_(C_14_H_16_N_3_O_4_)_2_(ClO_4_)_2_(C_10_H_8_N_2_)_2_]	*Z* = 1
*M**_r_* = 1390.79	*F*(000) = 716
Triclinic, *P*1	*D*_x_ = 1.689 Mg m^−^^3^
Hall symbol: -P 1	Mo *K*α radiation, λ = 0.71073 Å
*a* = 10.854 (4) Å	Cell parameters from 3928 reflections
*b* = 11.309 (4) Å	θ = 2.2–28.1°
*c* = 12.728 (5) Å	µ = 1.54 mm^−^^1^
α = 67.724 (4)°	*T* = 298 K
β = 73.357 (4)°	Block, green
γ = 75.411 (4)°	0.21 × 0.16 × 0.14 mm
*V* = 1367.0 (9) Å^3^	

### Data collection

**Table:**

Bruker SMART CCD diffractometer	4850 independent reflections
Radiation source: fine-focus sealed tube	3963 reflections with *I* > 2σ(*I*)
graphite	*R*_int_ = 0.015
φ and ω scans	θ_max_ = 25.2°, θ_min_ = 1.8°
Absorption correction: multi-scan (*SADABS*; Sheldrick, 1996)	*h* = −12→13
*T*_min_ = 0.738, *T*_max_ = 0.814	*k* = −13→9
7296 measured reflections	*l* = −15→12

### Refinement

**Table:**

Refinement on *F*^2^	Primary atom site location: structure-invariant direct methods
Least-squares matrix: full	Secondary atom site location: difference Fourier map
*R*[*F*^2^ > 2σ(*F*^2^)] = 0.032	Hydrogen site location: inferred from neighbouring sites
*wR*(*F*^2^) = 0.087	H-atom parameters constrained
*S* = 1.04	*w* = 1/[σ^2^(*F*_o_^2^) + (0.0445*P*)^2^ + 0.6427*P*] where *P* = (*F*_o_^2^ + 2*F*_c_^2^)/3
4850 reflections	(Δ/σ)_max_ = 0.001
383 parameters	Δρ_max_ = 0.33 e Å^−^^3^
0 restraints	Δρ_min_ = −0.56 e Å^−^^3^

### Special details

**Table:**

Experimental. Yield, 61%, analysis, calculated for C_50_H_56_Cl_2_N_10_O_18_Ni_4_: C 43.18, H, 4.06; N 10.07%; found: C 43.22, H 4.15, N, 10.09%.
Geometry. All esds (except the esd in the dihedral angle between two l.s. planes) are estimated using the full covariance matrix. The cell esds are taken into account individually in the estimation of esds in distances, angles and torsion angles; correlations between esds in cell parameters are only used when they are defined by crystal symmetry. An approximate (isotropic) treatment of cell esds is used for estimating esds involving l.s. planes.
Refinement. Refinement of F^2^ against ALL reflections. The weighted R-factor wR and goodness of fit S are based on F^2^, conventional R-factors R are based on F, with F set to zero for negative F^2^. The threshold expression of F^2^ > 2sigma(F^2^) is used only for calculating R-factors(gt) etc. and is not relevant to the choice of reflections for refinement. R-factors based on F^2^ are statistically about twice as large as those based on F, and R- factors based on ALL data will be even larger.

### Fractional atomic coordinates and isotropic or equivalent isotropic displacement parameters (Å^2^)

**Table:**

	*x*	*y*	*z*	*U*_iso_*/*U*_eq_	
Ni1	0.84712 (3)	−0.11565 (3)	0.73827 (3)	0.03336 (11)	
Ni2	0.72954 (3)	0.35869 (3)	0.46787 (3)	0.03195 (11)	
O1	0.9263 (2)	−0.2738 (2)	0.70987 (18)	0.0586 (6)	
O2	1.06150 (19)	−0.39494 (18)	0.61253 (18)	0.0456 (5)	
O3	0.7747 (2)	0.18517 (17)	0.45514 (16)	0.0400 (5)	
O4	0.73515 (19)	0.26666 (17)	0.63062 (16)	0.0380 (4)	
N1	0.8309 (2)	−0.0280 (2)	0.57245 (18)	0.0318 (5)	
N2	0.7889 (2)	0.0574 (2)	0.74936 (19)	0.0388 (5)	
N3	0.8932 (2)	−0.2005 (2)	0.9003 (2)	0.0437 (6)	
N4	0.6571 (2)	0.4495 (2)	0.32480 (19)	0.0367 (5)	
N5	0.6681 (2)	0.5300 (2)	0.4873 (2)	0.0363 (5)	
C1	0.9762 (3)	−0.2980 (3)	0.6150 (3)	0.0384 (6)	
C2	0.9259 (3)	−0.2161 (3)	0.5073 (2)	0.0338 (6)	
C3	0.9484 (3)	−0.2750 (3)	0.4233 (3)	0.0420 (7)	
H3	0.9979	−0.3573	0.4349	0.050*	
C4	0.8995 (3)	−0.2150 (3)	0.3240 (3)	0.0480 (7)	
H4	0.9160	−0.2555	0.2689	0.058*	
C5	0.8253 (3)	−0.0928 (3)	0.3077 (3)	0.0458 (7)	
H5	0.7905	−0.0514	0.2416	0.055*	
C6	0.8026 (3)	−0.0321 (3)	0.3886 (2)	0.0407 (7)	
H6	0.7522	0.0498	0.3761	0.049*	
C7	0.8531 (3)	−0.0904 (2)	0.4887 (2)	0.0324 (6)	
C8	0.7944 (2)	0.0971 (2)	0.5502 (2)	0.0309 (6)	
C9	0.7718 (3)	0.1443 (2)	0.6517 (2)	0.0330 (6)	
C10	0.7595 (4)	0.0988 (3)	0.8514 (3)	0.0522 (8)	
H10A	0.6682	0.0971	0.8886	0.063*	
H10B	0.7746	0.1873	0.8263	0.063*	
C11	0.8409 (4)	0.0144 (3)	0.9376 (3)	0.0613 (10)	
H11A	0.8175	0.0462	1.0028	0.074*	
H11B	0.9316	0.0221	0.9016	0.074*	
C12	0.8276 (4)	−0.1261 (3)	0.9829 (3)	0.0593 (9)	
H12A	0.7355	−0.1321	1.0053	0.071*	
H12B	0.8628	−0.1677	1.0525	0.071*	
C13	0.8543 (4)	−0.3307 (3)	0.9596 (3)	0.0563 (9)	
H13A	0.9003	−0.3859	0.9142	0.084*	
H13B	0.7622	−0.3233	0.9678	0.084*	
H13C	0.8754	−0.3673	1.0350	0.084*	
C14	1.0355 (3)	−0.2162 (4)	0.8801 (3)	0.0675 (10)	
H14A	1.0617	−0.2516	0.9533	0.101*	
H14B	1.0630	−0.1335	0.8377	0.101*	
H14C	1.0750	−0.2738	0.8359	0.101*	
C15	0.6502 (3)	0.3972 (3)	0.2487 (2)	0.0437 (7)	
H15	0.6840	0.3101	0.2603	0.052*	
C16	0.5948 (3)	0.4677 (3)	0.1537 (3)	0.0520 (8)	
H16	0.5908	0.4289	0.1023	0.062*	
C17	0.5453 (3)	0.5971 (3)	0.1365 (3)	0.0534 (8)	
H17	0.5081	0.6469	0.0728	0.064*	
C18	0.5513 (3)	0.6523 (3)	0.2147 (3)	0.0462 (7)	
H18	0.5187	0.7393	0.2042	0.055*	
C19	0.6065 (3)	0.5760 (3)	0.3084 (2)	0.0356 (6)	
C20	0.6125 (3)	0.6218 (3)	0.4019 (2)	0.0351 (6)	
C21	0.5636 (3)	0.7460 (3)	0.4056 (3)	0.0445 (7)	
H21	0.5251	0.8083	0.3467	0.053*	
C22	0.5732 (3)	0.7761 (3)	0.4990 (3)	0.0488 (8)	
H22	0.5405	0.8588	0.5033	0.059*	
C23	0.6312 (3)	0.6828 (3)	0.5847 (3)	0.0455 (7)	
H23	0.6387	0.7015	0.6476	0.055*	
C24	0.6781 (3)	0.5609 (3)	0.5758 (3)	0.0426 (7)	
H24	0.7182	0.4980	0.6334	0.051*	
Cl1	0.49509 (8)	−0.11510 (8)	0.81502 (7)	0.0508 (2)	
O5	0.6183 (3)	−0.1925 (3)	0.8306 (3)	0.0928 (10)	
O6	0.4947 (4)	−0.0733 (3)	0.6956 (3)	0.1125 (13)	
O7	0.4736 (3)	−0.0026 (3)	0.8472 (3)	0.0911 (9)	
O8	0.3950 (3)	−0.1885 (3)	0.8805 (4)	0.1155 (13)	
O9	0.9657 (4)	0.4193 (3)	0.8309 (3)	0.1074 (12)	
H9	0.9999	0.4646	0.7666	0.129*	
C25	0.8308 (6)	0.4297 (5)	0.8360 (5)	0.123 (2)	
H25A	0.7916	0.3737	0.9099	0.185*	
H25B	0.8209	0.4049	0.7750	0.185*	
H25C	0.7889	0.5176	0.8267	0.185*	

### Atomic displacement parameters (Å^2^)

**Table:**

	*U*^11^	*U*^22^	*U*^33^	*U*^12^	*U*^13^	*U*^23^
Ni1	0.0426 (2)	0.02493 (19)	0.02760 (19)	0.00177 (15)	−0.00980 (15)	−0.00623 (14)
Ni2	0.0383 (2)	0.02207 (18)	0.03246 (19)	0.00241 (14)	−0.01060 (15)	−0.00812 (14)
O1	0.0859 (17)	0.0354 (11)	0.0383 (12)	0.0159 (11)	−0.0141 (11)	−0.0103 (10)
O2	0.0396 (11)	0.0332 (11)	0.0550 (13)	0.0053 (9)	−0.0080 (9)	−0.0134 (9)
O3	0.0553 (13)	0.0289 (10)	0.0339 (10)	−0.0008 (9)	−0.0151 (9)	−0.0080 (8)
O4	0.0462 (12)	0.0275 (10)	0.0374 (10)	0.0018 (8)	−0.0103 (9)	−0.0114 (8)
N1	0.0363 (12)	0.0278 (11)	0.0302 (11)	−0.0024 (10)	−0.0077 (9)	−0.0098 (9)
N2	0.0522 (15)	0.0323 (12)	0.0298 (12)	−0.0015 (11)	−0.0115 (11)	−0.0094 (10)
N3	0.0489 (15)	0.0396 (14)	0.0371 (13)	−0.0032 (11)	−0.0124 (11)	−0.0068 (11)
N4	0.0383 (13)	0.0326 (12)	0.0367 (12)	−0.0034 (10)	−0.0083 (10)	−0.0101 (10)
N5	0.0352 (13)	0.0308 (12)	0.0393 (13)	−0.0027 (10)	−0.0064 (10)	−0.0105 (10)
C1	0.0363 (16)	0.0316 (15)	0.0440 (17)	−0.0056 (13)	−0.0044 (13)	−0.0120 (13)
C2	0.0291 (14)	0.0335 (14)	0.0389 (15)	−0.0065 (11)	−0.0028 (11)	−0.0144 (12)
C3	0.0401 (16)	0.0387 (16)	0.0489 (18)	−0.0033 (13)	−0.0038 (13)	−0.0225 (14)
C4	0.0510 (19)	0.0539 (19)	0.0478 (18)	−0.0093 (15)	−0.0067 (15)	−0.0286 (15)
C5	0.0536 (19)	0.0483 (18)	0.0398 (16)	−0.0132 (15)	−0.0133 (14)	−0.0136 (14)
C6	0.0465 (17)	0.0340 (15)	0.0421 (16)	−0.0040 (13)	−0.0137 (13)	−0.0116 (13)
C7	0.0313 (14)	0.0316 (14)	0.0332 (14)	−0.0073 (11)	−0.0028 (11)	−0.0111 (11)
C8	0.0307 (14)	0.0284 (13)	0.0306 (14)	−0.0015 (11)	−0.0075 (11)	−0.0079 (11)
C9	0.0320 (14)	0.0290 (14)	0.0363 (15)	−0.0030 (11)	−0.0072 (11)	−0.0103 (12)
C10	0.078 (2)	0.0426 (17)	0.0389 (17)	0.0012 (16)	−0.0177 (16)	−0.0195 (14)
C11	0.087 (3)	0.058 (2)	0.0435 (19)	0.0010 (19)	−0.0245 (18)	−0.0219 (16)
C12	0.081 (3)	0.055 (2)	0.0368 (17)	−0.0009 (18)	−0.0176 (17)	−0.0119 (15)
C13	0.068 (2)	0.0456 (19)	0.0420 (18)	−0.0086 (17)	−0.0132 (16)	0.0011 (14)
C14	0.049 (2)	0.077 (3)	0.066 (2)	−0.0087 (19)	−0.0188 (18)	−0.008 (2)
C15	0.0498 (18)	0.0396 (16)	0.0407 (16)	−0.0056 (14)	−0.0082 (14)	−0.0143 (13)
C16	0.062 (2)	0.055 (2)	0.0385 (17)	−0.0106 (17)	−0.0110 (15)	−0.0139 (15)
C17	0.054 (2)	0.059 (2)	0.0398 (17)	−0.0039 (16)	−0.0160 (15)	−0.0065 (15)
C18	0.0439 (18)	0.0388 (16)	0.0423 (17)	0.0022 (13)	−0.0104 (14)	−0.0034 (13)
C19	0.0296 (14)	0.0309 (14)	0.0390 (15)	−0.0020 (11)	−0.0046 (12)	−0.0073 (12)
C20	0.0302 (14)	0.0286 (14)	0.0414 (15)	−0.0033 (11)	−0.0060 (12)	−0.0079 (12)
C21	0.0414 (17)	0.0295 (15)	0.0558 (19)	−0.0017 (13)	−0.0088 (14)	−0.0105 (14)
C22	0.0462 (18)	0.0321 (16)	0.065 (2)	−0.0060 (14)	−0.0026 (15)	−0.0196 (15)
C23	0.0443 (17)	0.0427 (17)	0.0530 (19)	−0.0094 (14)	−0.0046 (14)	−0.0223 (15)
C24	0.0446 (17)	0.0414 (16)	0.0444 (17)	−0.0040 (13)	−0.0116 (14)	−0.0175 (14)
Cl1	0.0531 (5)	0.0441 (4)	0.0561 (5)	−0.0004 (4)	−0.0177 (4)	−0.0178 (4)
O5	0.0651 (18)	0.0728 (19)	0.117 (2)	0.0084 (15)	−0.0425 (17)	−0.0015 (17)
O6	0.156 (3)	0.104 (2)	0.074 (2)	0.046 (2)	−0.059 (2)	−0.0422 (19)
O7	0.122 (3)	0.0686 (18)	0.098 (2)	−0.0174 (17)	−0.0168 (19)	−0.0475 (17)
O8	0.081 (2)	0.076 (2)	0.181 (4)	−0.0366 (18)	0.016 (2)	−0.050 (2)
O9	0.180 (4)	0.0602 (19)	0.067 (2)	0.002 (2)	−0.036 (2)	−0.0106 (15)
C25	0.185 (6)	0.079 (3)	0.143 (5)	−0.048 (4)	−0.117 (5)	0.000 (3)

### Geometric parameters (Å, °)

**Table:**

Ni1—O1	1.899 (2)	C10—H10B	0.9700
Ni1—N2	1.945 (2)	C11—C12	1.500 (5)
Ni1—N1	2.002 (2)	C11—H11A	0.9700
Ni1—N3	2.067 (2)	C11—H11B	0.9700
Ni2—O4	1.9452 (19)	C12—H12A	0.9700
Ni2—O3	1.957 (2)	C12—H12B	0.9700
Ni2—N5	1.969 (2)	C13—H13A	0.9600
Ni2—N4	1.996 (2)	C13—H13B	0.9600
Ni2—O2^i^	2.276 (2)	C13—H13C	0.9600
O1—C1	1.276 (3)	C14—H14A	0.9600
O2—C1	1.248 (3)	C14—H14B	0.9600
O2—Ni2^i^	2.276 (2)	C14—H14C	0.9600
O3—C8	1.276 (3)	C15—C16	1.380 (4)
O4—C9	1.284 (3)	C15—H15	0.9300
N1—C8	1.310 (3)	C16—C17	1.381 (5)
N1—C7	1.426 (3)	C16—H16	0.9300
N2—C9	1.288 (3)	C17—C18	1.384 (4)
N2—C10	1.469 (4)	C17—H17	0.9300
N3—C14	1.468 (4)	C18—C19	1.376 (4)
N3—C13	1.484 (4)	C18—H18	0.9300
N3—C12	1.504 (4)	C19—C20	1.488 (4)
N4—C15	1.338 (4)	C20—C21	1.385 (4)
N4—C19	1.355 (3)	C21—C22	1.390 (4)
N5—C24	1.339 (4)	C21—H21	0.9300
N5—C20	1.351 (3)	C22—C23	1.373 (4)
C1—C2	1.498 (4)	C22—H22	0.9300
C2—C3	1.400 (4)	C23—C24	1.380 (4)
C2—C7	1.409 (4)	C23—H23	0.9300
C3—C4	1.375 (4)	C24—H24	0.9300
C3—H3	0.9300	Cl1—O8	1.405 (3)
C4—C5	1.386 (4)	Cl1—O6	1.411 (3)
C4—H4	0.9300	Cl1—O5	1.422 (3)
C5—C6	1.376 (4)	Cl1—O7	1.426 (3)
C5—H5	0.9300	O9—C25	1.424 (6)
C6—C7	1.393 (4)	O9—H9	0.8200
C6—H6	0.9300	C25—H25A	0.9600
C8—C9	1.513 (4)	C25—H25B	0.9600
C10—C11	1.494 (4)	C25—H25C	0.9600
C10—H10A	0.9700		
			
O1—Ni1—N2	171.10 (10)	C11—C10—H10B	109.2
O1—Ni1—N1	91.11 (9)	H10A—C10—H10B	107.9
N2—Ni1—N1	84.53 (9)	C10—C11—C12	114.6 (3)
O1—Ni1—N3	87.70 (10)	C10—C11—H11A	108.6
N2—Ni1—N3	95.41 (10)	C12—C11—H11A	108.6
N1—Ni1—N3	171.13 (10)	C10—C11—H11B	108.6
O4—Ni2—O3	84.13 (8)	C12—C11—H11B	108.6
O4—Ni2—N5	94.63 (9)	H11A—C11—H11B	107.6
O3—Ni2—N5	174.87 (9)	C11—C12—N3	115.9 (3)
O4—Ni2—N4	159.66 (9)	C11—C12—H12A	108.3
O3—Ni2—N4	97.63 (9)	N3—C12—H12A	108.3
N5—Ni2—N4	81.80 (9)	C11—C12—H12B	108.3
O4—Ni2—O2^i^	102.30 (8)	N3—C12—H12B	108.3
O3—Ni2—O2^i^	91.41 (8)	H12A—C12—H12B	107.4
N5—Ni2—O2^i^	93.72 (8)	N3—C13—H13A	109.5
N4—Ni2—O2^i^	97.92 (9)	N3—C13—H13B	109.5
C1—O1—Ni1	131.07 (19)	H13A—C13—H13B	109.5
C1—O2—Ni2^i^	116.82 (18)	N3—C13—H13C	109.5
C8—O3—Ni2	112.51 (16)	H13A—C13—H13C	109.5
C9—O4—Ni2	112.20 (17)	H13B—C13—H13C	109.5
C8—N1—C7	123.5 (2)	N3—C14—H14A	109.5
C8—N1—Ni1	110.62 (17)	N3—C14—H14B	109.5
C7—N1—Ni1	125.87 (17)	H14A—C14—H14B	109.5
C9—N2—C10	118.0 (2)	N3—C14—H14C	109.5
C9—N2—Ni1	112.66 (18)	H14A—C14—H14C	109.5
C10—N2—Ni1	129.33 (18)	H14B—C14—H14C	109.5
C14—N3—C13	108.2 (3)	N4—C15—C16	122.5 (3)
C14—N3—C12	111.1 (3)	N4—C15—H15	118.8
C13—N3—C12	105.6 (3)	C16—C15—H15	118.8
C14—N3—Ni1	106.2 (2)	C17—C16—C15	118.5 (3)
C13—N3—Ni1	111.19 (19)	C17—C16—H16	120.7
C12—N3—Ni1	114.47 (19)	C15—C16—H16	120.7
C15—N4—C19	118.7 (2)	C16—C17—C18	119.6 (3)
C15—N4—Ni2	126.8 (2)	C16—C17—H17	120.2
C19—N4—Ni2	114.46 (19)	C18—C17—H17	120.2
C24—N5—C20	119.1 (2)	C19—C18—C17	118.9 (3)
C24—N5—Ni2	125.47 (19)	C19—C18—H18	120.6
C20—N5—Ni2	115.39 (19)	C17—C18—H18	120.6
O2—C1—O1	120.4 (3)	N4—C19—C18	121.8 (3)
O2—C1—C2	119.2 (3)	N4—C19—C20	114.1 (2)
O1—C1—C2	120.3 (2)	C18—C19—C20	124.1 (3)
C3—C2—C7	119.0 (3)	N5—C20—C21	121.3 (3)
C3—C2—C1	115.6 (2)	N5—C20—C19	114.3 (2)
C7—C2—C1	125.3 (2)	C21—C20—C19	124.4 (3)
C4—C3—C2	121.9 (3)	C20—C21—C22	118.9 (3)
C4—C3—H3	119.0	C20—C21—H21	120.6
C2—C3—H3	119.0	C22—C21—H21	120.6
C3—C4—C5	118.7 (3)	C23—C22—C21	119.6 (3)
C3—C4—H4	120.7	C23—C22—H22	120.2
C5—C4—H4	120.7	C21—C22—H22	120.2
C6—C5—C4	120.6 (3)	C22—C23—C24	118.7 (3)
C6—C5—H5	119.7	C22—C23—H23	120.7
C4—C5—H5	119.7	C24—C23—H23	120.7
C5—C6—C7	121.6 (3)	N5—C24—C23	122.4 (3)
C5—C6—H6	119.2	N5—C24—H24	118.8
C7—C6—H6	119.2	C23—C24—H24	118.8
C6—C7—C2	118.2 (2)	O8—Cl1—O6	110.1 (3)
C6—C7—N1	122.0 (2)	O8—Cl1—O5	110.0 (2)
C2—C7—N1	119.8 (2)	O6—Cl1—O5	107.5 (2)
O3—C8—N1	129.3 (2)	O8—Cl1—O7	109.6 (2)
O3—C8—C9	115.1 (2)	O6—Cl1—O7	107.6 (2)
N1—C8—C9	115.5 (2)	O5—Cl1—O7	112.0 (2)
O4—C9—N2	127.6 (2)	C25—O9—H9	109.5
O4—C9—C8	115.8 (2)	O9—C25—H25A	109.5
N2—C9—C8	116.6 (2)	O9—C25—H25B	109.5
N2—C10—C11	112.3 (3)	H25A—C25—H25B	109.5
N2—C10—H10A	109.2	O9—C25—H25C	109.5
C11—C10—H10A	109.2	H25A—C25—H25C	109.5
N2—C10—H10B	109.2	H25B—C25—H25C	109.5

Symmetry codes: (i) −*x*+2, −*y*, −*z*+1.

### Hydrogen-bond geometry (Å, °)

**Table:**

*D*—H···*A*	*D*—H	H···*A*	*D*···*A*	*D*—H···*A*
O9—H9···O1^ii^	0.82	2.73	3.190 (4)	118
O9—H9···O2^ii^	0.82	2.06	2.870 (4)	170
C4—H4···O9^i^	0.93	2.52	3.387 (5)	155
C13—H13C···O9^iii^	0.96	2.58	3.451 (5)	151
C3—H3···O2	0.93	2.41	2.744 (4)	101
C6—H6···O3	0.93	2.22	2.812 (3)	121
C10—H10B···O4	0.97	2.43	2.775 (4)	100
C13—H13A···O1	0.96	2.39	2.899 (4)	113
C13—H13B···O5	0.96	2.47	3.186 (4)	131
C14—H14C···O1	0.96	2.58	3.074 (5)	112
C24—H24···O4	0.93	2.59	3.065 (4)	112

Symmetry codes: (ii) *x*, *y*+1, *z*; (i) −*x*+2, −*y*, −*z*+1; (iii) −*x*+2, −*y*, −*z*+2.

## References

[bb1] Bruker, (1998). *SMART* and *SAINT*. Bruker AXS, Madison, Wisconsin, USA.

[bb2] Sheldrick, G. M. (1996). *SADABS*. University of Göttingen, Germany.

[bb3] Sheldrick, G. M. (2008). *Acta Cryst.* A**64**, 112–122. 10.1107/S0108767307043930 18156677

[bb4] Spek, A. L. (2009). *Acta Cryst.* D**65**, 148–155. 10.1107/S090744490804362X PMC2631630 19171970

[bb5] Tao, R. J., Zang, S. Q., Cheng, Y. X., Wang, Q. L., Hu, N. H., Niu, J. Y. & Liao, D. Z. (2003). *Polyhedron* **22**, 2911–2916.

